# Coffee Infusions: Can They Be a Source of Microelements with Antioxidant Properties?

**DOI:** 10.3390/antiox10111709

**Published:** 2021-10-27

**Authors:** Ewa Olechno, Anna Puścion-Jakubik, Katarzyna Socha, Małgorzata Elżbieta Zujko

**Affiliations:** 1 Department of Food Biotechnology, Faculty of Health Science, Medical University of Białystok, Szpitalna 37 Street, 15-295 Białystok, Poland; ewa.olechno@sd.umb.edu.pl (E.O.); malgorzata.zujko@umb.edu.pl (M.E.Z.); 2 Department of Bromatology, Faculty of Pharmacy with the Division of Laboratory Medicine, Medical University of Białystok, Mickiewicza 2D Street, 15-222 Białystok, Poland; katarzyna.socha@umb.edu.pl

**Keywords:** coffee, chromium, cobalt, copper, fluoride, iron, manganese, zinc

## Abstract

Coffee is a beverage that is very popular all over the world. Its pro-health effect has been demonstrated in many publications. This drink can counteract the effects of oxidative stress thanks to its antioxidant properties. The aim of this study was to collect data on the content of microelements with antioxidant activity (manganese, zinc, copper, iron) in coffee infusions, taking into account various factors. The study considered publications from the years 2000–2020 found in Google Scholar and PubMed databases. It was noted that coffee can provide up to 13.7% of manganese requirements per serving, up to 4.0% and 3.1% of zinc requirements for women and men, up to 2.7% and 2.1% of copper requirements for women and men, and up to 0.4% and 0.6% of iron requirements for women and men. Coffee infusions can also be a source of fluoride (up to 2.5%), chromium (up to 0.4% of daily intake for women and 0.2% for men), and cobalt (up to 0.1%). There are no data in the literature regarding the content of selenium in coffee infusions. The origin of coffee beans and the type of water used (especially regarding fluoride) may have an impact on the content of minerals in infusions. The brewing method does not seem to play an important role. As it is a very popular beverage, coffee can additionally enrich the diet with such micronutrients as manganese, zinc, and copper. This seems beneficial due to their antioxidant properties, however the bioavailability of these elements of coffee should be taken into account. It seems necessary to carry out more research in this area.

## 1. Introduction

Coffee is a drink that is readily consumed all over the world, so it is important to carefully study its chemical composition. The main commercial species of coffee are Arabica (*Coffea arabica* L.) and Robusta (*Coffea canephora* Pierre ex Froehner) [1]. The largest consumers are Europeans, who in 2020/2021 drank 3243.9 tons of coffee—an increase of 0.5% compared to 2017/2018 [2]. Over the years, coffee has ceased to be treated as a stimulant and came to be seen as a drink with health-promoting properties, as shown in many studies [3,4,5,6,7,8]. Its positive effect is mainly attributed to polyphenols [9]. The reviewed research reveals that coffee is the main source of these compounds in many countries, including Poland, USA, Japan, and Brazil [10,11,12,13]. Other ingredients included in coffee beans are carbohydrates, proteins, fats, alkaloids, diterpenes, free amino acids, melanoidins formed during the roasting process, as well as minerals—both macro- and microelements [14]. Coffee is not usually presented as a source of minerals, but due to the fact that it is consumed frequently, it can also supplement some minerals. It has been shown that this drink can be a source of potassium and magnesium [15,16,17]. In addition to macronutrients, it contains certain amounts of microelements [18]. These ingredients are equally important, despite the fact that the human requirement for them is below 100 mg per day [19]. Microelements are involved in many biochemical processes that take place in the body; particularly important are those with antioxidant properties—zinc, manganese, copper, selenium, and iron [20]. They support various mechanisms and counteract the effects of oxidative stress [21]. Oxidative stress contributes to, among other things, lipid peroxidation and protein and DNA damage. It is important to ensure an adequate amount of microelements in the diet [22]. Taking into account the sources of antioxidants, we can divide them into exogenous and endogenous ones [23,24]. The latter arise naturally in our body. These include enzymes, such as superoxide dismutase (SOD) or glutathione peroxidase, as well as glutathione, coenzyme Q10, and N-acetylcysteine. Exogenous antioxidants are delivered with food. Among them are the abovementioned microelements, but also vitamins C, E, β-carotene and other carotenoids and polyphenolic compounds, including flavonoids and proanthocyanidins [23]. The sources of these substances in the daily diet are plant products, such as vegetables, fruits, legumes, groats, oils, nuts and seeds, as well as cocoa, herbs, coffee, or tea [25,26,27].

The content of minerals in beans, and thus coffee infusions, depends mainly on the origin of the coffee, which influences the natural content of a given element in the soil. Soil contamination can be caused by, among others, production processes, methods of soil cultivation including the use of fertilizers, as well as climatic conditions [16,28,29,30,31,32,33,34,35]. Brewing methods also play a vital role. We can distinguish, e.g., pour-over coffee, espresso, the drip method, the Turkish method, and the coffee percolator. They differ in terms of the amount of water and coffee used, the ratio of coffee to water, extraction time, water temperature, degree of coffee grinding, or the application of pressure [36]. The human body’s absorption of ingredients from infusions, i.e., bioavailability, should also be taken into account [37]. Since water consumed also contains minerals, it is difficult to assess the extent to which coffee supplies them, and the question requires further research [38].

**Figure 1:**
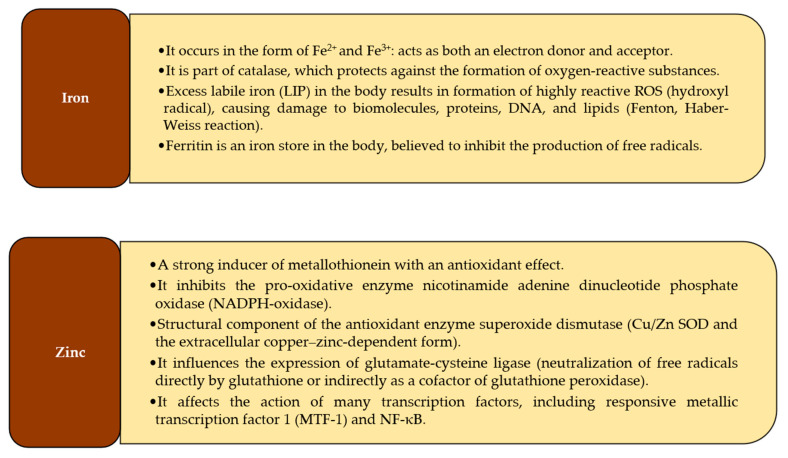

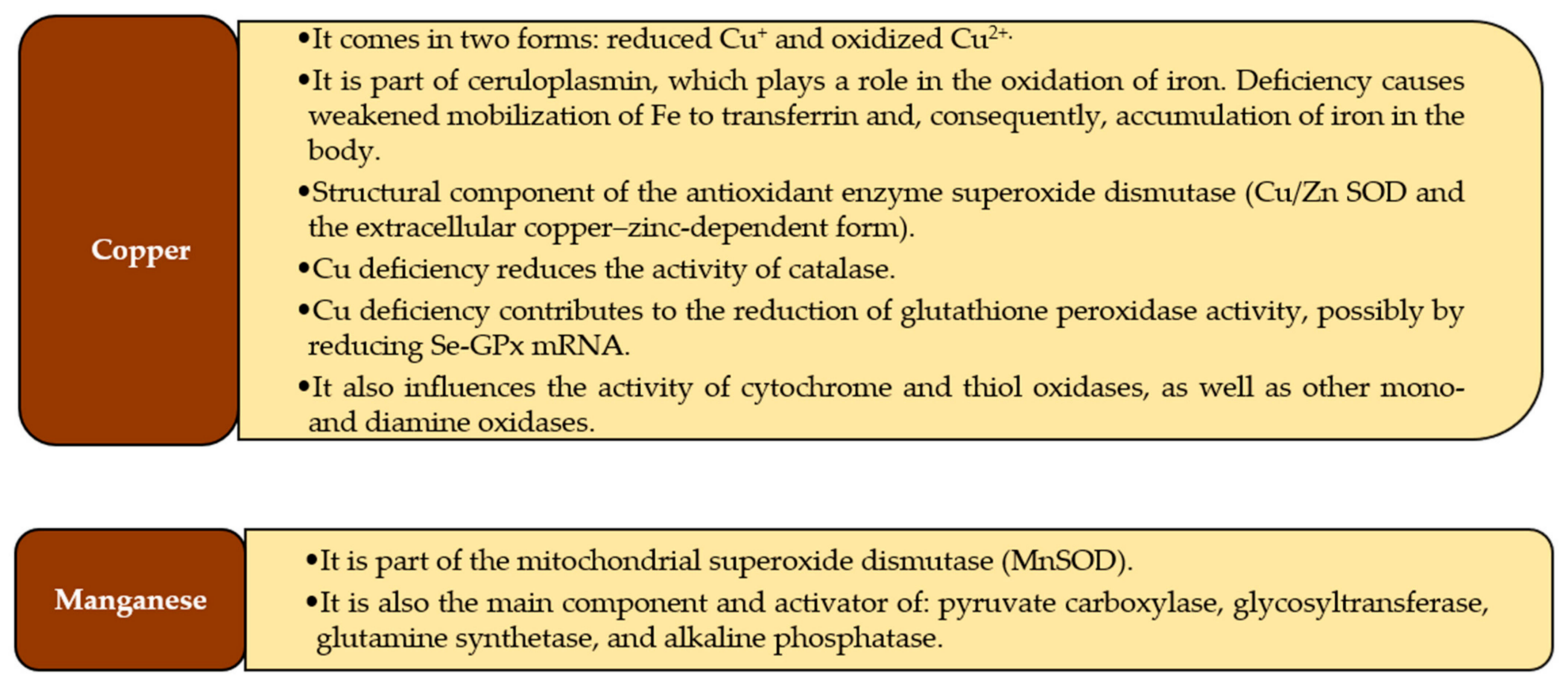
Summary of the most important antioxidant properties of microelements [39,40,41,42,43,44,45,46,47,48,49,50,51,52,53,54,55,56,57,58].

Microelements, such as iron, zinc, copper, and manganese, have antioxidant properties, as shown in Figure 1.

This publication focuses on studies assessing the content of antioxidant microelements in coffee infusions, as well as other microelements, and aims to determine whether coffee may be one of the sources of these elements.

## 2. Materials and Methods

The study considered papers from 2000–2020. The databases Google Scholar and PubMed were searched for the following phrases: ‘coffee’, ‘Arabica’, ‘Robusta’, ‘minerals in coffee brews’, ‘microelements in coffee brews’, ‘iron’, ‘zinc’, ‘copper’, ‘manganese’, ‘chromium’, ‘cobalt’, ‘fluorine’, and ‘minerals in water’. The analyzed publications included: specific type of coffee, brewing method used by consumers, brewing time, amount of coffee and water, cup volume, water temperature, water pressure, time and degree of coffee grinding, degree of roasting, and the origin of the coffee. The exclusion criterion was lack of information about the brewing method used by the consumer.

## 3. Results and Discussion

Tables 1–7 contain the literature data obtained as part of the review of traditional coffee infusion studies. In order to compare the results according to different authors, the method of presenting the content of individual minerals in infusions was standardized and converted, and then expressed in µg/100 mL. In some cases, the results (marked with ‘*’) were expressed on a ‘per 100 g’ basis to make them more comparable. It would have been necessary to determine the density of the brews prepared by the authors in the publications where the results were expressed as per 100 g in order to correctly assess the amounts of elements that they provide. Thus, these values were only referred to at the end of each paragraph. If the authors did not provide a conversion for daily requirement, we made one independently.

### 3.1. Zinc

Zinc is an element that performs a number of functions in the human body. It plays an important role in the activation of over 300 enzymes [47]. It influences the efficiency of the immune system, as well as the sense of taste and smell, fertility, and proper development of children [49,59]. It has also been shown to have a positive effect on increasing insulin sensitivity and improving glycemia [31]. Zinc has a crucial role in maintaining the so-called redox balance in cells. It is an inducer of metallothionein, which influences the antioxidant effect [60,61]. Moreover, it competes with pro-oxidative metals (iron and copper), which prevents the formation of reactive oxygen species [62]. Zinc is also a cofactor of many antioxidant enzymes, inhibits the pro-oxidative enzyme nicotinamide adenine dinucleotide phosphate oxidase (NADPH-Oxidase), and is part of the superoxide dismutase (Zn/Cu SOD), which is responsible for the neutralization of superoxide radicals [48,50]. On the other hand, excess zinc may reduce the expression of copper-dependent enzymes, including ceruloplasmin [59]. The extraction efficiency of zinc from roasted and ground coffee ranges from 22.3% to 61.5% [63,64,65], depending, to a large extent, on the formation of connections with polyphenols, alkaloids, or other compounds [66].

The content of zinc in the discussed infusions (Table 1) ranged from 8.0 ± 2.9 µg/100 mL for Turkish coffee (*Arabica*) in the study by Adler et al. (2019) [65] to 292.0 ± 80.0 µg/100 mL in coffee made with the same method (without a specific species) in the study by Özdestan (2013) [67]. The ratio of coffee to the amount of water in Adler et al. (2019) [65] was lower than in Özdestan (2013) [67], therefore it seems that it could have had a significant impact on the obtained zinc concentration. The beans of both coffees had been ground immediately before preparing the infusions. The differences may be due to the origin of the beans (undefined in both studies), the infusion time (undefined in Özdestan (2013) [67]), and the type of water (undefined in Adler et al. (2019) [65]). These factors can have an impact on the amount of mineral in the coffee brew.

In the case of pour-over coffee brews, the highest mean zinc content, expressed per unit volume (100 mL), was recorded by da Silva et al. (2016) [68]: 26 µg/100 mL, with the highest result in this study being 258.0 µg/100 mL of infusion. A similar value was found by Ashu and Chandravansh (2011) [64]: from 21.0 ± 0.8 to 30.0 ± 1.2 µg/100 mL (no species). The lowest zinc concentration using the same brewing method was obtained by Janda et al. (2020) [15]: about 13.5 µg/100 mL. These differences may be due to the different amounts of coffee and water used: 6 g of coffee/200 mL of water in Ashu and Chandravansh (2011) [64], 17 g/250 mL in Janda (2020) [15], 6 g/150 mL in Gogoaşă et al. (2016) [69], and 12 g/100 mL in da Silva et al. (2016) [68]. Da Silva et al. (2016) [68] used the largest amount of coffee in relation to the amount of water and thus obtained the highest concentration of this element. Janda et al. (2020) [15], despite a higher coffee/water ratio than Ashu and Chandravansh (2011) [64] and Gogoaşă et al. (2016) [69], recorded a lower concentration of zinc. It seems, therefore, that this factor could have played a role but was not necessarily decisive. In the case of zinc, the type of water can be important. It is worth emphasizing that the presence of zinc in drinking water is not desirable and the amount above 100 µg/L in tap water may result from the use of galvanized materials in older buildings [70]. Stelmach et al. (2013) [38] checked the effect of the type of water and noted that the highest concentration of zinc occurred in tap water, and the lowest in mineral water. No information of the type of water was provided by Ashu and Chandravansh (2011) [64] or da Silva et al. (2016) [68]. The temperature of water used by the researchers ranged from 92 to 100 °C. Such slight variations do not appear to be of significance in extracting the mineral. As shown by Stelmach et al. (2013) [38], the lowest concentration of zinc was found in an infusion at a temperature of 60–70 °C. On the other hand, an increase to 80 °C caused a rise in zinc concentration by 20%. Further increasing the temperature did not boost extraction. As mentioned earlier, the degree of grinding was defined only in Janda et al. (2020) [15] as very finely ground. All used coffees had been ground immediately prior to brewing, therefore zinc content could not have been influenced by grinding during the production process or contact with technological materials, according to Świetlik and Trojanowska (2014) [71]. The degree of roasting was not determined in the studies, and the origin (Ethiopia) was only given by Ashu and Chandravansh (2011) [64]. Ethiopian coffees differ in mineral composition depending on the variety [34], and the coffee used in that study was a blend of three different brands. Moreover, as research has shown, zinc is not abundantly present in Ethiopian coffees [72,73], while differences between zinc content in coffee from individual countries are noticeable [74].

Ground coffee brewed in an espresso machine proved to have from 17.0–23.0 µg/100 mL of zinc in Świetlik and Trojanowska (2014) [71] to 23.5 mg/100 mL in Janda et al. (2020) [15]. The ratio of coffee to water, as mentioned earlier, was probably higher in the Świetlik and Trojanowska study (2014) [71]. Additionally, neither study took into account the origin or degree of roasting. On the other hand, Świetlik and Trojanowska (2014) [71] did not provide information on the type of water and the pressure used. Differences may be due to various factors, but the origin of the coffee beans used should have been considered, which the authors neglected to do. Ground coffee prepared in a filter coffee machine in the Świetlik and Trojanowska study (2014) [71] had a higher concentration of zinc: 26–35 µg/100 mL of infusion than coffee from an espresso machine. As already mentioned, the authors emphasized that this method might favor the extraction of minerals, including zinc [71]. On the other hand, in the same study coffee made in a filter coffee machine from freshly ground Arabica had a lower zinc content (15.0–25.0 µg/100 mL) than a ground coffee infusion, which may have resulted from contamination during the production process [71]. The other variables used were the same. In the other brewing methods in Janda et al. (2020) [15], zinc concentrations ranged from about 12.5 µg/100 mL for the French press method to about 13.0 µg/100 mL for the drip method. Comparing all the brewing methods in this study, the most zinc was extracted in espresso, suggesting that this method may enhance the extraction of this element.

As for the concentration of the element expressed per 100 g, in the study by Stelmach et al. (2013) [38], pour-over coffee made from a mixture of Arabica and Robusta had a slightly higher average zinc concentration than pure Arabica, 70.0 µg and 6.0 µg/100 g of infusion, respectively. However, taking into account all ground coffee infusions prepared in this study, the Arabica infusion contained the highest concentration of zinc: 156.0 µg/100 g of infusion. As mentioned earlier, differences between the individual infusions may depend, among other things, on the ratio of coffee to the amount of water, type of water used, degree of grinding, roasting and origin of the coffee, and the growing and environmental conditions.

Calculations were made on the basis of publications in which the findings were presented per liter or milliliter of infusion. The infusions presented by weight were compared separately in order to make the comparisons meaningful. The infusion portions adopted when calculating the daily requirement were kept, as in the first part of the work, at 30 mL for espresso and 150 mL for other methods (pour-over coffee, Turkish method, drip method, French press, Aeropress). The volume of 150 mL was chosen to better visualize the obtained results.

The demand for zinc according to EFSA was established depending on the consumption of phytates in the range of 300–1200 mg/day, which makes absorption difficult. Given a phytate intake of 900 mg/day, the daily supply of zinc should be 11 mg/day for women and 14 mg/day for men [75]. In the case of the infusion of Turkish brewed coffee, which was found to have both the highest and the lowest zinc content, consumption of a cup of infusion provided 12–438 µg/150 mL, which covers 0.11–3.98% of women’s and 0.09–3.13% of men’s demand for this element. Consumption of pour-over ground coffee can provide 20–40 µg of zinc per cup (150 mL), which covers 0.18–0.36% of the requirement for this element in women and 0.14–0.29% in men [15,64,68]. An espresso infusion (30 mL) can provide 5–7 µg of zinc, i.e., 0.05–0.06% (women) and 0.04–0.05% (men) of the daily requirement for this element [15,71]. Pour-over coffee from the study by Stelmach et al. (2013) [38] would cover approximately 0.82–0.95% of the demand for zinc in women and 0.64–0.75% in men. It follows that coffee provides little or no zinc and should not be considered a source of zinc. Moreover, the bioavailability of this element is low. It is believed that its absorption, as in the case of iron, may be reduced by the formation of complexes with polyphenols [76,77,78].

### 3.2. Copper

Copper occurs in the body in either a reduced (Cu I) or oxidized (Cu II) form. Therefore, it plays the role of a cofactor of antioxidant enzymes, including cytochrome c oxidase, superoxide dismutase, lysyl oxidase, or tyrosinase [50]. It is part of ceruloplasmin, which catalyzes the oxidation of iron [55]. It also participates in the production of energy in cells, the production of melanin, myelin, hemoglobin, as well as the proper functioning of the thyroid gland [54,59,75]. The main sources of copper are grain products and meat [75]. Copper is an element that does not readily pass into coffee brew. It has been shown that coffee components form complexes with copper, making it difficult for it to be released into the brew [79]. The extraction efficiency of copper is low and amounts to 3.28–10.3% [63,64,65].

In the analyzed papers (Table 2), copper content ranged from 0.4 µg/100 mL in pour-over coffee (roasted coffee, Arabica) in da Silva et al. (2016) [68] to an average of 23.0 µg/100 mL also in pour-over coffee (green coffee, Robusta) in Jeszka-Skowron et al. (2016) [80], with the highest result in this study found in an infusion of green coffee (Robusta) from Laos, respectively: 34.4 µg/100 mL. In two brewing methods, Aeropress and the drip method in Janda et al. (2020) [15], no copper was detected. In the remaining studies, pour-over roasted coffee had lower copper concentrations than green coffee: about 2.0 µg/100 mL in Janda et al. (2020) [15], 2.1 µg/100 mL in Ashu and Chandravansh (2011) [64], and 2.7 µg/100 mL in Gogoaşă et al. (2016) [69]. The ratio of coffee to the amount of water was the highest in da Silva (2016) [68], 12 g/100 mL of water, and the lowest in Jeszka-Skowron (2016) [80]: 0.5 g/20 mL, which is inversely correlated with the copper concentrations obtained in these studies. It is worth mentioning that da Silva et al. (2016) [68] found copper in only one of the 50 types of Arabica coffee infusions tested. The higher coffee/water ratio in Janda et al. (2020) [15] than in Ashu and Chandravansh (2011) [64] and Gogoaşă et al. (2016) [69] was unlikely to have increased the concentration of copper in the infusion, as the obtained results did not differ significantly. It follows that this factor did not play a significant role in the case of this element. Moreover, in Jeszka-Skowron et al. (2016) [80], two species were considered: Arabica and Robusta. Copper content in Arabica is slightly higher than in Robusta, respectively: 15.0–21.0 µg/100 mL and 12.0–34.0 µg/100 mL. Earlier studies had noticed that, apart from manganese and phosphorus, copper could be a good element differentiating these varieties [81,82].

In the same study, the decaffeination process was also considered. This process removes caffeine from coffee beans. Today, decaffeination is carried out on green coffee beans with the use of solvents, such as water, ethyl acetate, dichloromethane, and supercritical carbon dioxide [83]. It seems that the loss of minerals could take place then, but as has been shown, the latest techniques do not cause significant losses of elements [31]. The decaffeination process has not been found to adversely affect the content of copper in Robusta infusions from Vietnam. The infusions contained the following amounts of the element: 19.0 µg/100 mL in a traditional green coffee infusion and 18.0 µg/100 mL in a decaffeinated infusion. These are not significant differences. However, there are differences in the extraction of minerals between green and roasted coffee, as demonstrated by Van Cuong et al. (2014) [84]. Green coffee in the study by Jeszka-Skowron et al. (2016) [80] had a higher concentration of copper than roasted coffees prepared with the same method, which is contrary to the study by Van Cuong et al. (2014) [84]. The authors of the latter study showed that the extraction of minerals was lower in green coffee compared to roasted coffee. These coffees, however, came from different regions, which could have been important. In two of the four analyzed studies, the type of water was not specified but, as shown by Stelmach et al. (2013) [38], it did not play a significant role in the extraction of copper. The temperature of the infusions ranged from 92 °C to 100 °C. As in the case of zinc, the aforementioned study showed an increase in copper extraction by about 30% at 80 °C, while leachability did not increase at higher temperatures [38]. The brewing time ranged from 5–10 min for roasted coffee to 15 min for green coffee in Jeszka-Skowron et al. (2020) [80]. Information about infusion time was not included in da Silva et al. (2016) [68]. As shown by Stelmach et al. (2013) [38], extending infusion time from 5 to 10 min may increase the concentration of copper in the infusion by up to 51%. Analysis of the collected data can lead to the assumption that extraction time could have had an impact on the obtained results due to the fact that the test with the longest infusion time yielded the highest copper content. However, comparing the study of Gogoaşă et al. (2016) [69] with a 10-min infusion time with the study by Ashu and Chandravansh (2011) [64] with a 5-min extraction time, no significant differences in the obtained results can be seen. Discussing the influence of the type of water is difficult due to the lack of information on this subject in the three studies. However, according to Stelmach et al. (2013) [38], the type of water—tap water, mineral water, and distilled water—does not affect copper content in infusions. It is worth considering the possibility of increased copper concentrations caused by contamination from materials containing this element [70]. The type of coffee was not included in every study, which makes it difficult to analyze the influence of this factor. The type of species does not seem to matter [31], as confirmed by Jeszka-Skowron et al. (2016) [80], who used Arabica and Robusta green coffee and noticed no significant difference between the species. When considering coffee grinding, it seems that the level of extraction of the substance into the brew should increase with the finer grinding of the beans. Among the coffees mixed with water, only Janda et al. (2020) [15] took into account the degree of grinding, and thus it is impossible to compare this feature with other results. The coffees used in the study by da Silva et al. (2016) [68] and Jeszka-Skowron et al. (2016) [80] were freshly ground, and no specific trend was observed, confirming the possible influence of processing on the content copper. Both the highest and the lowest contents of copper were found in beverages made from freshly ground coffee. Brews made from commercially ground coffee in Gogoaşă et al. (2016) [69] and Ashu and Chandravansh (2011) [64] had higher copper contents than those in da Silva et al. (2016) [68], but lower than in Jeszka-Skowron et al. (2016) [80].

The last two studies mentioned used freshly ground coffee. It can be concluded that there is no significant difference between freshly ground coffee beans and commercially ground coffee. However, according to Świetlik and Trojanowska (2014) [71], this effect may be visible in the case of prolonged contact of coffee during grinding with the elements of the machinery during the technological process. In such a case, ground coffee may have a higher content of a given element, which may have been present, for example, in the grinding blades [71]. The origin of coffee, and thus agrotechnical and environmental conditions, certainly have an impact, but there is not enough data on the origin in this study to compare this information. Copper content in coffee may be a feature that varies depending on the geographical origin of a given coffee [32]. For example, Ethiopian coffee has a higher copper content than Hawaiian [85] and Colombian coffees [86]. In Jeszka-Skowron et al. (2016) [80], it can be read that an Arabica infusion from China (Asia) had the lowest copper content, while a Robusta infusion with the highest concentration of this element came from Laos (Asia). There are significant differences regarding copper content in Arabica coffee from Brazil between da Silva et al. (2016) [68] and Jeszka-Skowron et al. (2016) [80]. According to dos Santos et al. (2010) [87], the content of copper largely depends on the way the land is cultivated, including the use of fertilizers.

As for coffee brewed in an espresso machine, its copper content ranged from 2.36 to 9.68 µg/100 mL in the study by Świetlik and Trojanowska (2014) [71] (no species) and amounted to 8.5 µg/100 mL in the study by Janda et al. (2020) [15] (Arabica). The ratio of coffee to water was probably higher in Świetlik and Trojanowska (2014) [71]. However, it does not seem to play an important role here, as only one brew in Świetlik and Trojanowska (2014) [71] had a higher concentration of copper than the infusion in Janda et al. (2020) [15]. The remaining factors for this method, due to insufficient data, were not analyzed.

On the other hand, when we compare coffee brewed in an espresso machine to that brewed in a filter coffee machine in the study by Świetlik and Trojanowska (2014) [71], both types of coffee contained similar amounts of copper. It follows that there are no significant differences between these brewing methods with regard to the extraction of copper. Moreover, in Świetlik and Trojanowska (2014) [71], freshly ground Arabica coffee prepared in a filter coffee machine had a medium concentration of copper: 5.08–6.53 µg/100 mL. However, this does not appear to be a decisive factor. When analyzing the influence of origin on the content of copper in an infusion from a filter coffee machine, it can be observed that freshly ground coffee from Sumatra was characterized by a slightly higher concentration of this element: 7 µg/100 mL [71].

The content of copper in Turkish brewed coffee was mentioned only in one study—Adler et al. (2019) [65]. The concentration of the element was 3.0 ± 2.0 µg/100 mL. This may be due to various factors. It seems that the method of brewing itself and the origin of the coffee were the most influential, but there is no research to compare this method in terms of copper content. It is worth mentioning that traditional crucibles for preparing Turkish coffee are made of copper, although today they are often made of stainless steel. Thus, copper from the pot could have found its way into the brew, but the above study did not take this factor into account. Janda et al. (2020) [15] compared several brewing methods and found the highest copper content in espresso (about 8.5 µg/100 mL) and French press coffee (about 8.0 µg/100 mL).

Considering copper content in ground coffee infusions presented per 100 g of infusion, again in the study by Stelmach et al. (2013) [38] pour-over mix of Arabica and Robusta contained a higher concentration of the element: 135 µg/100 g of infusion. According to Cruz et al. (2015) [31], Robusta may contain a slightly higher content of copper than Arabica, which could have influenced the obtained results.

The copper requirement is 1.3 mg/day for women and 1.6 mg/day for men [75]. The highest result in the discussed study for pour-over green coffee would provide 34.4 µg/150 mL of infusion (average: 23.0 µg/100 mL). This covers 2.69% of the demand for copper in women and 2.19% in men [80]. Pour-over roasted coffee had less copper: 1–5 µg/150 mL of infusion, which is 0.08–0.38% of the demand in women and 0.06–0.31% of the demand in men [15,64,68,69]. Stelmach et al. (2013) [38] recorded a high concentration of copper (per 150 g of infusion). The highest result for pour-over coffee would supply 15.6% of the demand of women and 12.7% of that of men. Espresso can provide 1.62–8.5 µg/30 mL, which covers 0.1–0.7% of the daily requirement of women and 0.1–0.5% of that of men [15,71]. Interestingly, Şemen et al. (2017) [88] also researched green coffee. According to the authors, the consumption of two cups of pour-over green coffee would provide a significant amount of copper: 2.94–7.37% of the daily requirement of 1 mg/day. On the other hand, Turkish brewed green coffee in the same study would cover as much as 5.37–9.94% of the norm for this element adopted by the authors [88]. For comparison, the amount of copper in the same method for roasted coffee in Adler et al. (2019) [65] would cover 0.45% of the daily copper requirement set at 1 mg, just as in the study by Şemen et al. (2017) [88]. It follows that green coffee may contain a greater amount of copper than roasted coffee. In summary, coffee infusions can be regarded as a source of copper. It is important that green coffee infusions provide more of this element. This could depend on the type of coffee used, with a naturally increased content, depending on the origin or on the differences in the extraction of green and roasted coffee. The matter certainly requires further research.

### 3.3. Manganese

Manganese is a microelement that participates in the transformation of amino acids, carbohydrates, and lipids [75,89]. It is also the main component and activator of the following enzymes: pyruvate carboxylase, glycosyltransferase, glutamine synthetase, alkaline phosphatase, and mitochondrial superoxide dismutase [56,57]. However, overexposure to manganese can be toxic. It contributes to the production of reactive oxygen species and toxic metabolites and influences the change of mitochondrial function and ATP production [89]. Manganese is found in tea, nuts, legumes, grain products, seafood, and to a lesser extent, in some vegetables and fruits [75], among others. The manganese extraction efficiency for ground and roasted coffee is in the range of 24.3% to 38.7% [63,64].

The content of manganese (Table 3) ranged from 15.0 µg/100 mL (average content, the lowest result: 5.0 µg/100 mL, Indonesia) for green pour-over coffee in the study by Jeszka-Skowron et al. (2016) [80] to 273.6 ± 71.1 µg/100 mL for Turkish brewed coffee in the study by Özdestan et al. (2013) [67].

Among the pour-over ground coffees, the highest concentration of manganese was found in an infusion of roasted Arabica in Janda et al. (2020) [15]: about 65.0 µg/100 mL. Green coffee in Jeszka-Skowron et al. (2016) [80] had a lower average concentration of manganese, while the highest result in this study was 75.0 µg/100 mL in green Arabica from China. Similarly, in da Silva et al. (2016) [68], the highest maximum result was 75 µg/100 mL. Da Silva et al. (2016) [68] used the highest coffee/water ratio, while the values for the 50 trials varied widely, the lowest being 26 µg/100 mL of brew. It follows that, depending on the origin of the coffee, different concentrations of the same element can be obtained using the same variables. The lowest coffee/water ratio was used by Jeszka-Skowron et al. (2016) [80]. In this study, the concentrations of the element also varied so it is not possible to draw an unequivocal conclusion as to the effect of the amount of coffee and water used. Infusion time was significantly longer in Jeszka-Skowron et al. (2016) [80] and amounted to 15 min. Da Silva et al. (2016) [68] did not specify this factor. In Janda et al. (2020) [15], where extraction lasted 5 min, a higher content of the element was detected than in Gogoaşă et al. (2016) [69] with a 10-min infusion time. Ashu and Chandravansh (2011) [64] obtained a similar mean manganese content despite a brewing time that was shorter by half than that used by Gogoaşă et al. (2016) [69]. Thus, another factor may have played a role.

The influence of the type of water is likely, but it seems that it is not the main factor influencing the content of manganese. Moreover, the type of water was not specified by da Silva et al. (2016) [68] or Ashu and Chandravansh (2011) [64]. As demonstrated by Stelmach et al. (2013) [38], the highest manganese extraction in the case of pour-over coffee occurs in distilled water. However, no particular tendency was noticed in the discussed studies. Jeszka-Skowron et al. (2016) [80], despite the use of distilled water, obtained very different results for individual coffees. It follows that another factor, such as the origin of the coffee, may have had an influence. Similarly, Janda et al. (2020) [15] used filtered water and obtained a higher concentration of manganese than Gogoaşă et al. (2016) [69] (distilled water), which is not in line with the conclusions of Stelmach et al. (2013) [38]. Another important factor is water temperature, which ranged from 92 °C in Janda et al. (2020) [15] to 100 °C in Ashu and Chandravansh (2011) [64] and Gogoaşă et al. (2016) [69]. These are not significant differences and, as noted above, increased manganese extraction into the infusion takes place already at 80 °C (a rise by about 10%), and the subsequent increase in temperature does not enhance extraction [38]. The leaching of the substance in the case of green coffee, as mentioned earlier, should theoretically be lower [84], which is at odds with the results obtained. However, in order to properly examine this relationship, it would be necessary to subject the used green coffee beans to the roasting process and prepare infusions in order to compare the effect of this factor. The effect of the time from coffee grinding does not appear to be significant in the case of manganese. The degree of grinding was determined only by Janda et al. (2020) [15]. In Jeszka-Skowron et al. (2016) [80], it can be noticed that manganese content in Arabica was slightly higher than in Robusta, respectively: 18.0–75.0 µg/100 mL and 5.2–25.4 µg/100 mL. Earlier studies noticed that Arabica contained more manganese than Robusta and it was found that it might be a distinguishing feature of particular varieties [81,82]. Decaffeination had no effect on the content of manganese in Robusta infusions. The infusions contained the following amounts: 16 µg/100 mL for traditional green coffee and 18 µg/100 mL for decaffeinated coffee. As in the case of copper, the differences are not significant [80]. As shown by Mehari et al. (2016) [32], the content of a given element may differ depending on the region of cultivation. Manganese has been recognized by some authors as an element that distinguishes Ethiopian coffee [32,74], as well as one that allows for evaluating the geographical origin of coffee beans from different countries around the world [74]. Habte et al. (2016) [73] noted that of all the microelements, manganese is found in the highest amounts in coffee beans from Ethiopia, respectively: Mn > Cu > Sr > Zn > Rb > Ni > B [73]. Ethiopian coffee may contain a slightly lower amount of manganese compared to Brazilian coffee [33]. However, the coffee brew from Ethiopia in Ashu and Chandravansh (2011) [64] did not receive the highest score. As emphasized by dos Santos et al. (2010) [87], the content of elements such as manganese in coffee beans, and thus in infusions, may depend on the use of fertilizers containing, among others, manganese, hence the differences between individual countries and regions. The use of coffees from different producers, despite the same origin of the coffee, as in the study by Debastiani et al. (2019) [79] where different brands of Brazilian coffees were used, also influences the content of elements, including manganese [79]. An infusion of Arabica from Brazil in Jeszka-Skowron et al. (2016) [80] contained half as much manganese as that described in da Silva et al. (2016) [68], respectively: 22.0 vs. 45.8 µg/100 mL. However, these studies differed as to the amount of coffee and water used. In Janda et al. (2020) [15], an espresso infusion contained about 50 µg/100 mL of manganese. Nevertheless, due to the lack of other studies that would take into account manganese content in this brewing method, it was not possible to discuss the individual features.

Manganese concentration in Turkish coffee in Özdestan et al. (2013) [67] averaged 273.6 ± 71.1 µg/100 mL. As with espresso, this is the only study to look at the content of manganese in coffee made with this brewing method. However, it can be concluded that the values differed significantly for the Turkish method, depending on the coffee brand, from 144.7 ± 3.0 to 414.6 ± 19.0 µg/100 mL. In the other brewing methods in Janda et al. (2020) [15], the concentrations of this element ranged from 44.3 µg/100 mL (French press) to about 64.0 µg/100 mL (Aeropress). Comparing all the brewing methods in this study, pour-over coffee and Aeropress coffee were the most efficient for extracting manganese.

Regarding manganese content in infusions of pour-over ground coffee presented per 100 g, Stelmach et al. (2013) [38] obtained a higher concentration of the element in Arabica than in a mixture of Arabica and Robusta, respectively: 1210 and 913 µg/100 g, which may confirm the conclusions drawn earlier by other authors [81,82]. On the other hand, Nędzarek et al. (2013) [90] recorded a lower result for pour-over coffee, from 497.0 ± 10.0 to 628.0 ± 101.0 µg/100 g of infusion, but the type of coffee was not specified [90]. The ratio of coffee to water was slightly lower in Stelmach et al. (2013) [38] than in Nędzarek et al. (2013) [90], although that did not affect the final result. It seems that the origin of the coffee, including the use of fertilizers, may have played a role [87]. In Oliveira et al. (2015) [16] (not included in the table due to non-compliance with the adopted inclusion criteria), manganese content in espresso infusions showed statistically significant differences (*p* < 0.05) among all the countries on different continents. It follows that it may be an element that indicates geographical origin. The highest average concentration of manganese was detected in infusions from South American coffee, while the lowest was in infusions from African coffee [16]. These differences may have resulted from both the natural content of the element in the soil and the use of fertilizers [87]. As demonstrated by Van Cuong et al. (2014) [84], manganese concentrations in coffee beans rose along with increasing temperature and roasting degree, and the highest was reached at 250 °C, which is probably related to water loss [84]. No particular tendency was observed for the Robusta admixture to contribute to significant differences in manganese concentrations. Two separate Arabica and Robusta infusions would have to be compared to observe any interspecies difference.

According to EFSA, the demand for manganese is 3.0 mg/day for people over the age of 25 [75]. An infusion of Turkish coffee with the highest concentration of this element would provide 414.6 ± 19.0 µg/150 mL, which is 13.7% of the daily requirement. A cup of pour-over green coffee would provide the lowest amount of manganese: 23 µg /150 mL of infusion, which is 0.8% of the recommended daily intake [67,80]. Infusions of roasted pour-over coffee (150 mL of infusion) would cover 1.0–3.3% [15,64,68,69] and 24.9–69.5% (150 g of infusion) of the daily norm for manganese [38,90]. On the other hand, the highest average result for green coffee in the same brewing method would provide 1.7% of the daily demand [80]. An espresso infusion (30 mL) in Janda et al. (2020) [15] would provide a slightly lower amount of manganese: 0.5% of the daily requirement. In another study, a cup of espresso would cover 1.4–1.9% of the daily norm for manganese, according to the standard adopted by the authors of the study (2 mg/day) [16]. As can be seen, a cup of coffee can be a source of manganese in the diet, but it is difficult to judge the decisive factors that play a role in the extraction of this nutrient. There is also a lack of research on the bioavailability of this element from coffee.

### 3.4. Iron

Iron is a microelement responsible for oxygen transport (in hemoglobin and myoglobin), energy metabolism, and electron transfer. It is part of the catalase oxidative enzyme, and therefore is a very important element in the fight against free radicals, provided that it is present in the right amount in the body. This element occurs in two oxidation states: in the form of Fe^2+^ and Fe^3+^ ions, so it can be both an acceptor and an electron donor. Fe^3+^ ions can be reduced to Fe^2+^ ions in the presence of reducing agents, for instance superoxide radical anions. Fe^2+^ ions catalyze the formation of dangerous hydroxyl radicals from hydrogen peroxide [39,41]. Iron can be divided into heme (in animal products) and non-heme iron (in plant products) [75]. The former is much better absorbed and is found mainly in red meat and offal, poultry, fish, and egg yolks, while non-heme iron is present in cereal products, beans, nuts, dark green vegetables, and enriched foods [91,92]. The extraction efficiency for iron in the case of roasted and ground coffee is only 4.9–13.2% [63,64,65]. It is an element with which coffee components can enter into complexes and thus ground coffee can ‘absorb’ some of the iron, according to Debastiani et al. (2019) [79].

The content of iron (Table 4) in the infusions in question ranged from an average of 15.8 µg/100 mL for Turkish brewed coffee in the study by Gogoaşă et al. (2016) [69] (no species) to about 43.9 µg/100 mL in Janda et al. (2020) [15] (Arabica). In Janda et al. (2020) [15], three brewing methods (drip method, espresso, and simple infusion) yielded the same result. Due to the lack of other studies analyzing iron that would take into account Turkish coffee and the drip method, it was only possible to compare the above types of infusions. These are two different brewing methods, but it can be seen that the coffee/water ratio was higher in the drip method [15], 18 g/300 mL of water, than in the Turkish coffee method, 10 g/200 mL [65]. The infusion time was half as short in Adler et al. (2019) [65]: 5 min. Different types of water and water temperature (92 °C and 100 °C) were also used. The origin of coffee was not taken into account in either study. Coffee in Janda et al. (2020) [15] was commercially ground, while Adler et al. (2019) [65] used fresh ground coffee, which could have influenced the content of iron. According to Świetlik and Trojanowska (2014) [71], higher iron content in ground coffee may result from abrasion of metal elements during the grinding of commercially produced coffee [71]. Moreover, a higher iron content may be the result of contamination of coffee beans with soil. According to Tagliaferro et al. (2007) [93], washed grains have a much lower content of this element than unwashed ones. The same relationship was noticed in espresso infusions made from these beans. Iron can be considered an element that indicates soil contamination of coffee beans [93]. In other studies using pour-over ground coffee, researchers obtained lower values for this method than Janda et al. (2020) [15]: 18.3 µg/100 mL in Ashu and Chandravansh (2011) [64] and 15.8 µg/100 mL in Gogoaşă et al. (2016) [69]. The preparation of the infusions varied. In Janda et al. (2020) [15], the highest coffee/water ratio was used and the highest iron content was obtained among pour-over ground coffees, while Gogoaşă et al. (2016) [69] used the lowest coffee/water ratio and obtained the lowest iron concentration. Infusion times were 5 min in Janda et al. (2020) [15] and 10 min in Gogoaşă et al. (2016) [69]. In Gogoaşă et al. (2016) [69], despite the longest infusion time, the final iron concentration was again the lowest. The temperature of water was 92 °C in Janda et al. (2020) [15] and 100 °C in Ashu and Chandravansh (2011) [64] and Gogoaşă et al. (2016) [69]. According to Stelmach et al. (2013) [38], the highest iron concentration can be observed in water temperatures of 80 °C, while at 100 °C there is a decrease by 10% (maximum). It stems from the above that the lower temperature in Janda et al. (2020) [15] may have been one of the factors which impacted the result. The type of water may also have had an influence on the extraction of iron.

Stelmach et al. (2013) [38] noticed that an infusion prepared from distilled water had the highest concentration of iron, while one made from mineral water had the lowest. Ashu and Chandravansh (2011) [64] did not specify the type of water, therefore it is difficult to assess its influence. On the other hand, the concentration of iron was lower in Gogoaşă et al. (2016) [69] where distilled water was used than in Janda et al. (2020) [15], where filtered water was used. It is worth noting, however, that some iron ions may remain in filtered water, and the number of these ions may even increase after using a jug filter [94], therefore it is not possible to notice a specific trend. Taking into account iron content in espresso in the study by Janda et al. (2020) [15], the concentration of this element was higher than in the study by Świetlik and Trojanowska (2014) [71]: 18.7–33.9 µg/100 mL. The ratio of coffee to the amount of water was not determined because Janda et al. (2020) [15] only gave the obtained volume of infusion, while Świetlik and Trojanowska (2014) [71] specified the amount of water used. However, it can be assumed that the coffee/water ratio was higher in Świetlik and Trojanowska (2014) [71]. The degree of roasting was not included in the discussed studies, while information on the degree of grinding was only provided by Janda et al. (2020) [15]. Origin could be an important factor, but it was not included in either of the studies. Coffee prepared in a filter coffee machine (Arabica) contained from 18.7 µg/100 mL (Sumatra) to 22.9 µg/100 mL (Ethiopia) in Świetlik and Trojanowska (2014) [71]. These values were lower than for coffee prepared in an espresso machine in the Janda (2020) study. However, as Anderson (2002) reports, coffee from Sumatra is characterized by a high iron content, while Ethiopian coffee has less of the element [95], in contrast to what Świetlik and Trojanowska (2014) [71] reported. It is worth noting that the coffee in Janda et al. (2020) [15] had been commercially ground (capsules), while Świetlik and Trojanowska (2014) [71] used freshly ground coffee. This may be a confirmation of the earlier assumptions of Świetlik and Trojanowska (2014) [71]. Additionally, in Świetlik and Trojanowska (2014) [71] the concentration of iron in ground coffee infusions (no species) was higher in filter coffee machine infusions (29–34 µg/100 mL) than in espresso machine coffee (19–26 µg/100 mL). The authors of the study concluded that the preparation of coffee in a filter coffee machine may be conducive to the extraction of iron. This may be due to a longer extraction time. Comparing all the brewing methods used in Janda et al. (2020) [15], the French press method contained the lowest iron concentration (about 35 µg/100 mL), while the highest results, as mentioned earlier, were obtained by three methods: drip method, espresso, and simple infusion. It follows that the choice of the brewing method for iron did not play a significant role as three completely different infusions had the same amount of the element.

Iron content presented per 100 g of infusion was the highest in an infusion of a mixture of Arabica and Robusta (pour-over) in Stelmach et al. (2013) [38] at 393.0 µg/100 g, while the lowest in Turkish brewed coffee (Arabica) in Fercan et al. (2016) [96] was 0.8 µg/100 g. Again, the differences may have resulted from the origin of the beans or the method of brewing, but also from the coffee/water ratio, which was the lowest in Fercan et al. (2016) [96]. The values in an infusion of Arabica ground coffee in Stelmach et al. (2013) [38] were lower than those in the Arabica and Robusta mix: 227.0 µg/100 g of infusion.

The iron requirement for healthy adult women (>25 years of age, premenopausal period) is 16 mg/day, and for men (>25 years of age) is 11 mg/day [75]. This amount increases in people with anemia, pregnant women, and women who are heavily menstruating, and decreases in postmenopausal women (up to 11 mg/day). The content of iron in a cup of infusion (150 mL) ranged from 20 µg in Turkish coffee [65] to 66 µg in coffee made by means of the drip method [15]. This is 0.13–0.41% of daily intake for women and 0.18–0.6% of daily intake for men. Pour-over ground coffee (150 mL) satisfied 0.15–0.41% of women’s needs and 0.22–0.6% of men’s needs for this element [15,64]. Espresso in the discussed studies would provide 0.04–0.41% of women’s needs and 0.09–0.6% of men’s needs [15,71]. According to Oliveira et al. (2015) [16], the consumption of a cup of espresso (5–6 g) would account for 0.07–0.15% of the daily norm for iron. The researchers assumed the daily requirement for this element at 14 mg/day [16]. In studies presented in grams of infusion, the highest result for pour-over coffee would cover 3.68% of the demand of women and 5.36% of the demand of men [38], with the lowest being for Turkish brewed coffee: 0.009% of the demand of women and 0.02% of the demand of men [96].

Coffee provides certain amounts of iron, but it is worth emphasizing that it is non-heme iron [97]. Additionally, phenolic compounds in coffee hinder the absorption of this element, possibly by forming complexes in the intestinal lumen [98]. Morck et al. (1983) [99] concluded that consumption of coffee with a meat meal reduced iron absorption by 39%. In addition, coffee consumption one hour before a meal had no effect on iron absorption, while its consumption one hour after a meal had the same negative effect as consumption during a meal [99]. Another study by Brune et al. (1989) [100] showed >60% inhibition of iron bioavailability from coffee. The authors considered chlorogenic acid. Layrisse et al. (2000) [101] noticed a 50% reduction in iron absorption when espresso was consumed with a meal, while the effect of Americana coffee was not observed, which the authors contributed to twice as high an amount of coffee in espresso. It can therefore be concluded that coffee can inhibit iron absorption and should not be considered a source of this element.

## 4. Other Microelements in Coffee Brews

### 4.1. Cobalt

Cobalt is widely distributed in the natural environment and has industrial applications. This element plays an important role in the human body as a component of vitamin B12 (cobalamin) and is responsible for the proper functioning of the nervous system and inter alia by participating in the creation of neurotransmitters [102]. It enters the body with food and through the respiratory system, skin, and biomaterials. Overexposure to cobalt can cause dangerous health effects, such as overproduction of red blood cells, asthma, and pulmonary fibrosis [103,104].

The values for cobalt (Table 5) ranged from 1.5 ± 0.1 µg/100 mL for all coffee brewing methods in Janda et al. (2020) [15] to 2.4 ± 0.1 µg/100 mL in pour-over coffee in Ashu and Chandravansh (2011) [64]. The ratio of coffee to water in the latter publication was significantly lower, while the infusions in both studies contained cobalt concentrations that did not differ significantly. As mentioned earlier, Ashu and Chandravansh (2011) [64] did not specify the type of water used. The brewing time did not differ, but the temperatures of the infusions slightly did. However, there is a lack of data in the literature on the effect of temperature on cobalt. It seems that the origin of the coffee, which was not mentioned by Janda et al. (2020) [15], could have had an impact. As shown, Ethiopia may have higher cobalt levels compared to other countries [33,74]. This may be due to the fact that Central Africa is rich in cobalt deposits, especially in Zambia and the Democratic Republic of Congo. However, Ethiopia is located in a different region. Environmental pollution with this element is taken into account [105,106].

Cobalt content per 100 g of pour-over ground coffee infusion in the study by Nędzarek et al. (2013) [90] ranged from 6.6 ± 0.7 to 7.0 ± 2.0 µg/100 g.

So far, no standard for cobalt has been specified. On the other hand, the lethal dose of LD50 was set at 150–500 mg/kg body weight [107]. However, as stated by Martin et al. (2019) [108], a harmful effect of cobalt on liver hepatocytes can occur at lower concentrations. In another study, the safely tolerated oral dose was established at 30 µg/kg/day [109]. Cobalt consumption varies across societies, ranging from 5 to 50 µg per day [110]. A study conducted in 2020 estimated that cobalt consumption by the Italian population amounted to 19 µg/day [111]. Based on the tolerable oral dose established by Finley et al. (2012) [109], for a person weighing 60 kg the daily safe intake of cobalt would be 1800 µg/day. An infusion of pour-over coffee (150 mL) with the highest result would cover only 0.17% of this dose, while with the lowest would be 0.08% of the norm [15,64]. The infusions described by Nędzarek et al. (2013) [90] (150 g) would provide 0.5–0.58% of the daily norm for cobalt. Semen et al. (2017) [88] determined that two cups of green coffee would provide as much as 20.9–49.8% of the cobalt requirement, set at 2 µg/day. Certainly, these results encourage further research in this direction. It should be emphasized that the amount of cobalt in an infusion may vary. This seems to be particularly dependent on the origin of the coffee. The inclusion of a coffee infusion in one’s diet should not pose a risk of exceeding the upper limit of safe intake of cobalt.

### 4.2. Chromium

Trivalent chromium is a trace element that is essential for the proper metabolism of carbohydrates, fats, and proteins. It is involved in glucose metabolism by influencing the action of insulin. The sources of chromium include: cereal products, egg yolks, nuts, broccoli, green beans, meat, yeast, and coffee [112].

The content of chromium (Table 6) described by the authors under investigation ranged from 0.228 µg/100 mL for espresso (no species) and for one of the infusions prepared in a drip coffee machine in the study by Świetlik and Trojanowska et al. (2014) [71] to 3.7 µg/100 mL for Arabica in the Aeropress method in Janda et al. (2020) [15]. Aeropress was only used by Janda et al. (2020) [15], therefore it is not possible to compare the factors in this brewing method. On the other hand, Janda et al. (2020) [15] also prepared an espresso coffee infusion, which had a slightly higher chromium content than that reported by Świetlik and Trojanowska (2014) [71], i.e., about 2.7 µg/100 mL. The ratio of coffee to water in Świetlik and Trojanowska (2014) [71] was significantly higher than in Janda et al. (2020) [15], assuming that the amount of water used in the latter study was similar to the given volume of infusion. Świetlik and Trojanowska (2014) [71] used 9 g of coffee/75 mL of water, while Janda et al. (2020) [15] was 17 g of coffee, and the volume of the brew was 250 mL. Moreover, in Świetlik and Trojanowska (2014) [71], the type of water was not specified, which could also, to some extent, have had an impact on the final chromium content. Water temperature in Janda et al. (2020) [15] was 92 °C. In Świetlik and Trojanowska (2014) [71] it was probably similar, as it is a characteristic temperature for infusions prepared in an espresso machine [113,114]. The authors of this study also did not specify the type of coffee, the pressure used, or the degree of grinding of the coffee. It seems that the last two factors may affect the extraction of minerals, while the type of coffee itself probably does not play a role in the case of chromium [31]. Neither of the studies mentioned the degree of roasting or the origin of the coffee. The place where coffee beans are harvested seems to be of considerable importance, as opposed to the degree of roasting [84].

No significant difference was noticed between coffee brewed in a filter coffee machine, whether commercially ground or freshly ground, and coffee made in an espresso machine in the same study [71]. In the case of freshly ground coffee brewed in a filter coffee machine, the highest concentration was detected in coffee from Ethiopia at 0.401 µg/100 mL, and significantly lower in coffee from Brazil and Sumatra at 0.183 µg/100 mL. In Gure et al. (2018) [74], a positive correlation of chromium and manganese in Ethiopian coffee was noticed, which may indicate a natural source or anthropogenic origin. However, these countries are characterized by neither significantly high nor significantly low content of these elements in coffee [74,95]. In pour-over coffee, chromium content was 2.2 µg/100 mL in da Silva [68] et al. (2017) and about 3.4 µg/100 mL in Janda et al. (2020) [15]. The ratio of the amount of coffee used to the amount of water was higher in da Silva et al. (2017) [68] at 12 g of coffee to 100 mL of water than in Janda et al. (2020) [15] at 17 g of coffee to 250 mL of water. However, the former infusion contained a lower concentration of chromium. Therefore, this factor did not play a significant role. Factors that may play a role are brewing time, type of water, and degree of grinding of the coffee beans—not specified in the study by da Silva et al. (2017) [68]—and the origin of coffee itself, not specified in either study. The degree of roasting and the similar water temperature did not seem to play a significant role. Other brewing methods in Janda et al. (2020) [15] (drip method and French press) produced a chromium content of about 3.3 and 2.7 µg/100 mL, respectively. As for extraction efficiency in the same study, espresso and simple infusion had the lowest chromium content, but no significant differences were found regarding the effect of individual brewing methods on the content of this element [15].

The average chromium content in infusions of pour-over ground coffee expressed per 100 g ranged from 3.5 ± 0.1 to 6.0 ± 4.0 µg/100 g of infusion in the study by Nędzarek et al. (2013) [90].

The demand for chromium has not been established by the EFSA [75]. The World Health Organization states that the average demand should be 0.025 mg/kg body weight/day for women and 0.035 mg/kg bw/day for men under 50 [70]. After conversion, the demand for chromium by a woman weighing 60 kg is 1.5 mg/day, and by a man weighing 70 kg is 2.45 mg/day. Consumption of 150 mL of coffee infusion with the highest result for the Aeropress method obtained in the discussed studies would cover 0.4% of the woman’s demand and 0.24% of the man’s demand for this element [15]. Coffee (150 mL) would provide 0.2–0.3% of the daily norm for a woman and 0.12–0.18 of the daily norm for a man [15,68]. Pour-over coffee (150 g) analyzed by Nędzarek et al. (2013) [90] would cover 0.4–0.75% of the woman’s demand and 0.24–0.37% of the man’s demand. Espresso (30 mL) would be a source of 0.004–0.06% of the daily dose of chromium in women and 0.002–0.04% of the daily dose in men [15,71]. The content of chromium in the coffee infusions in question was low.

### 4.3. Fluoride

According to the EFSA, fluoride is not an element necessary for the development of teeth, but it significantly reduces the risk of caries and protects teeth against the effects of acids. This element affects bone mineralization but, depending on the dose, it may have a positive or delayed effect. The main sources of fluoride are fluoride-containing water and fluoride-based drinks [75].

Fluoride content (Table 7) in the study by Wolska et al. (2017) [115] differed significantly, both between species and across methods of infusion. The authors examined the content of fluoride in Arabica and Robusta roasted coffee and in green coffee. They did not specify the type of water used, so it can be assumed that it was tap water, which significantly affects the concentration of fluoride in an infusion. The results were read from the graphs and are approximate. Arabica overflow espresso had the highest content of fluoride among roasted coffees, about 8 µg/100 mL. On the other hand, the lowest concentration of fluoride was found in Turkish-style brewed Robusta: about 1 µg/100 mL. A Robusta infusion had a higher concentration of the element in the simple infusion method and espresso maker (coffee percolator): 4 µg/100 mL (Robusta) and 3 µg/100 mL (Arabica) in simple infusion; 3.7 µg/100 mL (Robusta) and 2 µg/100 mL (Arabica) in coffee from the percolator.

An Arabica infusion proved to have noticeably higher content of fluoride in the case of overflow espresso and slightly higher in Turkish-style coffee: 7.5 (Arabica) and 2.0 µg/100 mL (Robusta) in the overflow espresso method, 2.2 µg/100 mL (Arabica) and 1.0 µg/100 mL (Robusta) in the Turkish coffee method. The other values were similar. The highest content of all tested coffees, both roasted and green, was detected in green coffee brewed using the Turkish method: about 50 µg/100 mL. Moreover, green coffee scored higher than roasted coffee in each brewing method in this study. The authors emphasized that roasting coffee might result in the formation of less soluble fluoride compounds, which are less able to be infused. There are no studies that checked the influence of infusion time on fluoride content in a brew, but it is assumed that the longer the time, the greater the content, as in the case of tea [116,117,118]. The content of fluoride in coffee is not high and is unlikely to be dangerous. Animal studies have shown that fluoride administered in coffee or caffeine solutions reaches higher levels in the body than when administered as plain water. This may be due to a temporary increase in the absorption rate, which may be related to caffeine content [119,120].

The requirement for fluoride was determined at 0.05 mg/kg body weight, as unanimously cited by all sources (not only dietary) [75]. Assuming a person weighs 60 kg, their requirement is 3 mg/day. An infusion of green coffee with the highest content of fluoride would cover 2.5% of the daily requirement for this element. By comparison, the maximum level of fluoride in roasted coffee would only cover 0.4% of the demand. The Robusta infusion with the lowest score would only cover 0.05% of the requirement. Accordingly, green coffee may be a better source of fluoride than roasted coffee. However, it should be remembered that this depends largely on the content of fluoride in the water, whereas the content of this element in the coffee infusion itself is difficult to estimate.

## 5. Conclusions

Coffee can enrich a diet with microelements that have antioxidant activity: manganese (up to 13.7% of the daily requirement), zinc (up to 4.0% of the daily requirement of women; 3.1% of the daily requirement of men), copper (up to 2.7% of the daily requirement of women; 2.1% of the daily requirement of men), and iron (up to 0.4% of the daily requirement of women; 0.6% of the daily requirement of men). Unfortunately, there are no studies that have investigated selenium content in coffee infusions. Coffee also provides some amounts of fluoride (up to 2.5%), chromium (up to 0.4 daily intake for women; 0.2% daily intake for men), and cobalt (up to 0.1%). However, there are significant discrepancies between the results obtained for manganese. Factors that could have influenced the outcomes are inter alia, the origin of coffee beans, including the natural content of a given element in the soil, and climatic and agrotechnical conditions. It seems that the type of water used to prepare infusions also plays an important role (especially in the case of fluoride), as does the type of brewing method, though to a lesser extent. In the case of green coffee, it can provide more copper and fluoride than roasted coffee. Perhaps this is due to the differences in the extraction of these ingredients between roasted and green coffee. Since coffee is widely consumed in many societies, it can be considered one of the sources of manganese, zinc, and copper, which seems beneficial due to their antioxidant properties. It is worth noting, however, that coffee can also hinder the absorption of zinc and iron, and this may exclude it as a source of these elements. There is a need for more research that would also take into account the content of individual minerals in the water used to prepare infusions, as well as their bioavailability for humans.

## Figures and Tables

**Table 1:** Zinc content in coffee infusions.

Content Av. ± SD (Min–Max) (µg/100 mL or 100 g)	Method of Brewing	Time (min)	Coffee (g)	Water (mL)	Cup Volume (mL)	Type of Water	Pressure (Ba)	Temperature of Water (°C)	Species	Degree of Roasting	Type of Coffee	Origin	Method of Analysis	Reference
292 ± 80	Turkish coffee	nd	5	65	nd	ultrapure distilled water	nd	nd	Arabica	roasted	fine ground	nd	HR-CS-FAAS	[67]
35	filter coffee machine	nd	9	75	nd	nd	nd	nd	nd	roasted	ground	nd	GF-AAS	[71]
29	filter coffee machine	nd	9	75	nd	nd	nd	nd	nd	roasted	ground	nd	GF-AAS	[71]
26.2 ± 11.8	pour-over	nd	12	100	nd	nd	nd	95–100	Arabica	medium roasted	fresh ground	Brazil (Cerrado Mineiro)	FS-FAAS	[68]
26	filter coffee machine	nd	9	75	nd	nd	nd	nd	nd	roasted	ground	nd	GF-AAS	[71]
25	filter coffee machine	nd	9	75	nd	nd	nd	nd	Arabica	roasted	fresh ground	Sumatra	GF-AAS	[71]
23.5	coffee machine	nd	17	nd	250	filtered	9	92	Arabica	roasted	fine ground	nd	ICP-OES	[15]
23	coffee machine	nd	9	75	nd	nd	nd	nd	nd	roasted	ground	nd	GF-AAS	[71]
22	coffee machine	nd	9	75	nd	nd	nd	nd	nd	roasted	ground	nd	GF-AAS	[71]
21.0 ± 0.9–30.0 ± 1.2	pour-over	5	6	200	nd	nd	nd	100	nd	nd	ground	Ethiopia	FAAS	[64]
17	coffee machine	nd	9	75	nd	nd	nd	nd	nd	roasted	ground	nd	GF-AAS	[71]
17	filter coffee machine	nd	9	75	nd	nd	nd	nd	Arabica	roasted	fresh ground	Brazil	GF-AAS	[71]
15	filter coffee machine	nd	9	75	nd	nd	nd	nd	Arabica	roasted	fresh ground	Ethiopia	GF-AAS	[71]
14.6 (12.4–16.3)	pour-over	10	6	150	nd	distilled water	nd	100	nd	nd	powder coffee	nd	FAAS	[69]
13.5	pour-over	5	17	250	nd	filtered	nd	92	Arabica	roasted	very fine ground	nd	ICP-OES	[15]
13	drip	2,5	18	300	nd	filtered	nd	92	Arabica	roasted	medium coarse ground	nd	ICP-OES	[15]
12.5	French press	5	17	300	nd	filtered	1–2	92	Arabica	roasted	medium ground	nd	ICP-OES	[15]
12.3	Aeropress	2	18	nd	250	filtered	2–4	93	Arabica	roasted	coarse ground	nd	ICP-OES	[15]
8 ± 2.9 (5.53–13.17)	Turkish coffee	5	10	200	nd	nd	nd	100	nd	nd	fresh ground	nd	FAAS	[65]
66 * (52–76)	pour-over	10	6	200	nd	re-distilled water	nd	100	Arabica & Robusta mix	roasted	ground	nd	FAAS	[38]
6 * (11–156)	pour-over	10	6	200	nd	re-distilled water	nd	100	Arabica	roasted	ground	nd	FAAS	[38]

FAAS—flame atomic absorption spectrometry, GF-AAS—graphite furnace atomic absorption spectrometry, HR-CS-FAAS—high-resolution source flame atomic absorption spectrometry, ICP-OES—inductively coupled plasma—optical emission spectrometry, nd—no data, * content in 100 g of brew.

**Table 2:** Copper content in coffee infusions.

Content Av. ± SD (Min–Max) (µg/100 mL or 100 g)	Method of Brewing	Time (min)	Coffee (g)	Water (mL)	Cup Volume (mL)	Type of Water	Pressure (Ba)	Temperature of Water (°C)	Species	Degree of Roasting	Type of Coffee	Origin	Method of Analysis	Reference
23 (11.5–34.4)	pour-over	15	0.5	20	nd	double distilled water	nd	95	Robusta	green	fresh ground	Vietnam, India Cherry Laos FAQ Indonesia Uganda SC12 Uganda Bugishu	ET AAS	[80]
18.1	pour-over	15	0.5	20	nd	double distilled water	nd	95	Robusta (decaffeinated coffee)	green	fresh ground	Vietnam	ET AAS	[80]
14.6–20.5	pour-over	15	0.5	20	nd	double distilled water	nd	95	Arabica	green	fresh ground	Brazil TG Rwanda Ordinary China Laos Guatemala Peru HB	ET AAS	[80]
9.68	coffee machine	nd	9	75	nd	nd	nd	nd	nd	roasted	ground	nd	GF-AAS	[71]
8.85	filter coffee machine	nd	9	75	nd	nd	nd	nd	nd	roasted	ground	nd	GF-AAS	[71]
8.5	coffee machine	nd	17	nd	250	filtered water	9	92	Arabica	roasted	fine ground	nd	ICP-OES	[15]
~8	French press	5	17	300	nd	filtered water	1–2	92	Arabica	roasted	medium ground	nd	ICP-OES	[15]
6.53	filter coffee machine	nd	9	75	nd	nd	nd	nd	Arabica	roasted	fresh ground	Sumatra	GF-AAS	[71]
5.63	filter coffee machine	nd	9	75	nd	nd	nd	nd	Arabica	roasted	fresh ground	Ethiopia	GF-AAS	[71]
5.08	filter coffee machine	nd	9	75	nd	nd	nd	nd	Arabica	roasted	fresh ground	Brazil	GF-AAS	[71]
3.04 ± 1.95 (1.20–6.86)	Turkish coffee	5	10	200	nd	nd	nd	100	nd	nd	fresh ground	nd	FAAS	[65]
2.7 (2.3–3.2)	pour-over	10	6	150	nd	distilled water	nd	100	nd	nd	powder coffee	nd	FAAS	[69]
2.53	filter coffee machine	nd	9	75	nd	nd	nd	nd	nd	roasted	ground	nd	GF-AAS	[71]
2.36	coffee machine	nd	9	75	nd	nd	nd	nd	nd	roasted	ground	nd	GF-AAS	[71]
2.1 ± 0.1–4.2 ± 0.6	pour-over	5	6	200	nd	nd	nd	100	nd	nd	ground	Ethiopia	FAAS	[64]
~2	pour-over	5	17	250	nd	filtered water	nd	92	Arabica	roasted	very fine ground	nd	ICP-OES	[15]
1.93	filter coffee machine	nd	9	75	nd	nd	nd	nd	nd	roasted	ground	nd	GF-AAS	[71]
1.62	coffee machine	nd	9	75	nd	nd	nd	nd	nd	roasted	ground	nd	GF-AAS	[71]
0.4	pour-over	nd	12	100	nd	nd	nd	95–100	Arabica	medium roasted	fresh ground	Brazil (Cerrado Mineiro)	FS-FAAS	[68]
below the detection limit	Aeropress	2	18	nd	250	filtered water	2–4	93	Arabica	roasted	coarse ground	nd	ICP-OES	[15]
below the detection limit	drip	2,5	18	300	nd	filtered water	nd	92	Arabica	roasted	medium coarse ground	nd	ICP-OES	[15]
135 * (112–165)	pour-over	10	6	200	nd	re-distilled water	nd	100	Arabica & Robusta mix	roasted	ground	nd	FAAS	[38]
<30–51 *	pour-over	10	6	200	nd	re-distilled water	nd	100	Arabica	roasted	ground	nd	FAAS	[38]

ET AAS—electro thermal atomic absorption spectrometry, FAAS—flame atomic absorption spectrometry, GF-AAS—graphite furnace atomic absorption spectrometry, ICP-OES—inductively coupled plasma—optical emission spectrometry, nd—no data, * content in 100 g of brew.

**Table 3:** Manganese content in coffee infusions.

Content Av. ± SD (Min–Max) (µg/100 mL or 100 g)	Method of Brewing	Time (min)	Coffee (g)	Water (mL)	Cup Volume (mL)	Type of Water	Pressure (Ba)	Temperature of Water (°C)	Species	Degree of Roasting	Type of Coffee	Origin	Method of Analysis	Reference
273.6 ± 71.1 (144.7 ± 3–414.6 ± 19)	Turkish coffee	nd	5	65	nd	ultrapure distilled water	nd	nd	Arabica	roasted	fine ground	nd	HR-CS-FAAS	[67]
~65	pour-over	5	17	250	nd	filtered water	nd	92	Arabica	roasted	very fine ground	nd	ICP-OES	[15]
~64	Aeropress	2	18	nd	250	filtered water	2–4	93	Arabica	roasted	coarse ground	nd	ICP-OES	[15]
~60	drip	2.5	18	300	nd	filtered water	nd	92	Arabica	roasted	medium coarse ground	nd	ICP-OES	[15]
~50	coffee machine	nd	17	nd	250	filtered water	9	92	Arabica	roasted	very fine ground	nd	ICP-OES	[15]
45.8	pour-over	nd	12	100	nd	nd	nd	95–100	Arabica	medium roasted	fresh ground	Brazil (Cerrado Mineiro)	FS-FAAS	[68]
44.3	French press	5	17	300	nd	filtered water	1–2	92	Arabica	roasted	medium ground	nd	ICP-OES	[15]
32.6 (18–75)	pour-over	15	0.5	20	nd	double distilled water	nd	95	Arabica	green	fresh ground	Brazil TG, Rwanda Ordinary, China, Laos, Guatemala Peru HB	ET AAS	[80]
28 ± 2 (18.9 ± 1.2–27.9 ± 1.5)	pour-over	5	6	200	nd	nd	nd	100	nd	nd	ground	Ethiopia	FAAS	[64]
20.6 (17.5–25.7)	pour-over	10	6	150	nd	distilled water	nd	100	nd	nd	powder coffee	nd	FAAS	[69]
17.6	pour-over	15	0.5	20	nd	double distilled water	nd	95	Robusta (decaffeinated coffee)	green	fresh ground	Vietnam	ET AAS	[80]
15 (5.2–25.4)	pour-over	15	0.5	20	nd	double distilled water	nd	95	Robusta	green	fresh ground	Vietnam, India Cherry Laos FAQ Indonesia Uganda SC12 Uganda Bugishu	ET AAS	[80]
1210 * (949–1460)	pour-over	10	6	200	nd	re-distilled water	nd	100	Arabica	roasted	ground	nd	FAAS	[38]
913 * (578–1330)	pour-over	10	6	200	nd	re-distilled water	nd	100	Arabica & Robusta mix	roasted	ground	nd	FAAS	[38]
628 ± 101 *	pour-over	nd	1	nd	27	nd	nd	nd	nd	roasted	ground	nd	ICP-MS	[90]
619 ± 2 *	pour-over	nd	1	nd	27	nd	nd	nd	nd	roasted	ground	nd	ICP-MS	[90]
502 ± 52 *	pour-over	nd	1	nd	27	nd	nd	nd	nd	roasted	ground	nd	ICP-MS	[90]
497 ± 10 *	pour-over	nd	1	nd	27	nd	nd	nd	nd	roasted	ground	nd	ICP-MS	[90]

ET AAS—electro thermal atomic absorption spectrometry, FAAS—flame atomic absorption spectrometry, FS-FAAS—fast sequential flame atomic absorption spectrometry, HR-CS-FAAS—high-resolution source flame atomic absorption spectrometry, ICP-MS—inductively coupled plasma mass spectrometry, ICP-OES—inductively coupled plasma—optical emission spectrometry, nd—no data, * content in 100 g of brew.

**Table 4:** Iron content in coffee infusions.

Content Av. ± SD (Min–Max) (µg/100 mL or 100 g)	Method of Brewing	Time (min)	Coffee (g)	Water (mL)	Cup Volume (mL)	Type of Water	Pressure (Ba)	Temperature of Water (°C)	Species	Degree of Roasting	Type of Coffee	Origin	Method of Analysis	Reference
~44	pour-over	5	17	250	nd	filtered	nd	92	Arabica	roasted	very fine ground	nd	ICP-OES	[15]
43.9	drip	2.5	18	300	nd	filtered	nd	92	Arabica	roasted	medium coarse ground	nd	ICP-OES	[15]
~43	coffee machine	nd	17	nd	250	filtered	9	92	Arabica	roasted	very fine ground	nd	ICP-OES	[15]
~42.5	Aeropress	2	18	-	250	filtered	2–4	93	Arabica	roasted	coarse ground	nd	ICP-OES	[15]
34.6	French press	5	17	300	nd	filtered	1–2	92	Arabica	roasted	medium ground	nd	ICP-OES	[15]
33.9	filter coffee machine	nd	9	75	nd	nd	nd	nd	nd	roasted	ground	nd	GF-AAS	[71]
28.7	filter coffee machine	nd	9	75	nd	nd	nd	nd	nd	roasted	ground	nd	GF-AAS	[71]
28.5	filter coffee machine	nd	9	75	nd	nd	nd	nd	nd	roasted	ground	nd	GF-AAS	[71]
26.2	coffee machine	nd	9	75	nd	nd	nd	nd	nd	roasted	ground	nd	GF-AAS	[71]
23.8	coffee machine	nd	9	75	nd	nd	nd	nd	nd	roasted	ground	nd	GF-AAS	[71]
22.9	filter coffee machine	nd	9	75	nd	nd	nd	nd	Arabica	roasted	fresh ground	Ethiopia	GF-AAS (3100 Perkin Elmer)	[71]
19.4	filter coffee machine	nd	9	75	nd	nd	nd	nd	Arabica	roasted	fresh ground	Brazil	GF-AAS	[71]
18.7	coffee machine	nd	9	75	nd	nd	nd	nd	nd	roasted	ground	nd	GF-AAS	[71]
18.7	filter coffee machine	nd	9	75	nd	nd	nd	nd	Arabica	roasted	fresh ground	Sumatra	GF-AAS	[71]
18.3 (13.8 ± 1.2–21.0 ± 1.8)	pour-over	5	6	200	nd	nd	nd	100	nd	nd	ground	Ethiopia	FAAS	[64]
15.8 (9.1–18.4)	pour-over	10	6	150	nd	distilled water	nd	100	nd	nd	powder coffee	nd	FAAS	[69]
393 * (322–430)	pour-over	10	6	200	nd	re-distilled water	nd	100	Arabica & Robusta mix	roasted	ground	nd	FAAS	[38]
227 * (113–324)	pour-over	10	6	200	nd	re-distilled water	nd	100	Arabica	roasted	ground	nd	FAAS	[38]
15.33 ± 533 * (8.93–24.50)	Turkish coffee	5	10 ± 0.1	200	nd	nd	nd	100	nd	nd	fresh ground	nd	FAAS	[65]
0.8 *	Turkish coffee	nd	2	100	nd	distilled water	nd	nd	Arabica	roasted	nd	nd	ICP-OES	[96]

FAAS—flame atomic absorption spectrometry, GF-AAS—graphite furnace atomic absorption spectrometry, ICP-OES—inductively coupled plasma—optical emission spectrometry, nd—no data, * content in 100 g of brew.

**Table 5:** Cobalt content in coffee infusions.

Content Av. ± SD (µg/100 mL or 100 g)	Method of Brewing	Time (min)	Coffee (g)	Water (mL)	Cup Volume (mL)	Type of Water	Pressure (Ba)	Temperature of Water (°C)	Species	Degree of Roasting	Type of Coffee	Origin	Method of Analysis	Reference
1.5 ± 0.1–2.4 ± 0.1	pour-over	5	6	200	nd	nd	nd	100	nd	nd	ground	Ethiopia	FAAS	[64]
1.2	pour-over	5	17	250	nd	filtered water	nd	92	Arabica	roasted	very fine ground	nd	ICP-OES	[15]
1.12	drip	2.5	18	300	nd	filtered water	nd	92	Arabica	roasted	medium coarse ground	nd	ICP-OES	[15]
0.9	Aeropress	2	18	nd	250	filtered water	2–4	93	Arabica	roasted	coarse ground	nd	ICP-OES	[15]
0.68	French press	5	17	300	nd	filtered water	1–2	92	Arabica	roasted	medium ground	nd	ICP-OES	[15]
0.6	coffee machine	nd	17	nd	250	filtered water	9	92	Arabica	roasted	very fine ground	nd	ICP-OES	[15]
7 ± 2 *	pour-over	nd	1	nd	27	nd	nd	nd	nd	roasted	ground	nd	ICP-MS	[90]
6.6 ± 0.7 *	pour-over	nd	1	nd	27	nd	nd	nd	nd	roasted	ground	nd	ICP-MS	[90]
6 ± 2 *	pour-over	nd	1	nd	27	nd	nd	nd	nd	roasted	ground	nd	ICP-MS	[90]
6 ± 2 *	pour-over	nd	1	nd	27	nd	nd	nd	nd	roasted	ground	nd	ICP-MS	[90]

FAAS—flame atomic absorption spectrometry, ICP-MS—inductively coupled plasma mass spectrometry, ICP-OES—inductively coupled plasma-optical emission spectrometry, nd—no data, * content in 100 g of brew.

**Table 6:** Chromium content in coffee infusions.

Content Av. ± SD (µg/100 mL or 100 g)	Method of Brewing	Time (min)	Coffee (g)	Water (mL)	Cup Volume (mL)	Type of Water	Pressure (Ba)	Temperature of Water (°C)	Species	Degree of Roasting	Type of Coffee	Origin	Method of Analysis	Reference
3.7	Aeropress	2	18	nd	250	filtered	2–4	93	Arabica	roasted	coarse ground	nd	ICP-OES	[15]
~3.4	pour-over	5	17	250	nd	filtered	nd	92	Arabica	roasted	very fine ground	nd	ICP-OES	[15]
~3.3	drip	2.5	18	300	nd	filtered	nd	92	Arabica	roasted	medium coarse ground	nd	ICP-OES	[15]
~3.2	French press	5	17	300	nd	filtered	1–2	92	Arabica	roasted	medium ground	nd	ICP-OES	[15]
~2.7	coffee machine	nd	17	nd	250	filtered	9	92	Arabica	roasted	very fine ground	nd	ICP-OES	[15]
2.2	pour-over	nd	12	100	nd	nd	nd	95–100	Arabica	medium roasted	fresh ground	Brazil (Cerrado Mineiro)	FS-FAAS	[68]
0.401	filter coffee machine	nd	9	75	nd	nd	nd	nd	Arabica	roasted	fresh ground	Ethiopia	GF-AAS	[71]
0.362	filter coffee machine	nd	9	75	nd	nd	nd	nd	nd	roasted	ground	nd	GF-AAS	[71]
0.291	coffee machine	nd	9	75	nd	nd	nd	nd	nd	roasted	ground	nd	GF-AAS	[71]
0.260	filter coffee machine	nd	9	75	nd	nd	nd	nd	nd	roasted	ground	nd	GF-AAS	[71]
0.228	filter coffee machine	nd	9	75	nd	nd	nd	nd	Arabica	roasted	fresh ground	Sumatra	GF-AAS	[71]
0.220	coffee machine	nd	9	75	nd	nd	nd	nd	nd	roasted	ground	nd	GF-AAS	[71]
0.211	filter coffee machine	nd	9	75	nd	nd	nd	nd	nd	roasted	ground	nd	GF-AAS	[71]
0.183	filter coffee machine	nd	9	75	nd	nd	nd	nd	Arabica	roasted	fresh ground	Brazil	GF-AAS	[71]
0.170	coffee machine	nd	9	75	nd	nd	nd	nd	nd	roasted	ground	nd	GF-AAS	[71]
6 ± 4 *	pour-over	nd	1	nd	27	nd	nd	nd	nd	roasted	ground	nd	ICP-MS	[90]
5 ± 2 *	pour-over	nd	1	nd	27	nd	nd	nd	nd	roasted	ground	nd	ICP-MS	[90]
4 ± 2 *	pour-over	nd	1	nd	27	nd	nd	nd	nd	roasted	ground	nd	ICP-MS	[90]
3.5 ± 0.1 *	pour-over	nd	1	nd	27	nd	nd	nd	nd	roasted	ground	nd	ICP-MS	[90]

FS-FAAS—fast sequential flame atomic absorption spectrometry, GF-AAS—graphite furnace atomic absorption spectrometry, ICP-MS—inductively coupled plasma mass spectrometry, ICP-OES—inductively coupled plasma-optical emission spectrometry, nd—no data, * content in 100 g of brew.

**Table 7:** Fluoride content in coffee infusions.

Content Av. ± SD (µg/100 mL or 100 g)	Method of Brewing	Time (min)	Coffee (g)	Water (mL)	Cup Volume (mL)	Type of Water	Pressure (Ba)	Temperature of Water (°C)	Species	Degree of Roasting	Type of Coffee	Origin	Method of Analysis	Reference
~50	Turkish coffee	5	1.5	150	nd	nd	nd	100	nd	green	fresh ground	Guatemala (Antigua region)	ISE	[115]
~14	coffee percolator	5	1.5	150	nd	nd	nd	100	nd	green	fresh ground	Guatemala (Antigua region)	ISE	[115]
~8	pour-over	5	1.5	150	nd	nd	nd	100	nd	green	fresh ground	Guatemala (Antigua region)	ISE	[115]
~7.5	filter coffee machine	5	1.5	150	nd	nd	nd	100	Arabica	roasted	fresh ground	Guatemala (Antigua region)	ISE	[115]
~7	filter coffee machine	5	1.5	150	nd	nd	nd	100	nd	green	fresh ground	Guatemala (Antigua region)	ISE	[115]
~4.7	French press	5	1.5	150	nd	nd	nd	100	nd	green	fresh ground	Guatemala (Antigua region)	ISE	[115]
~4	pour-over	5	1.5	150	nd	nd	nd	100	Robusta	roasted	fresh ground	India	ISE	[115]
~3.7	coffee percolator	5	1.5	150	nd	nd	nd	100	Robusta	roasted	fresh ground	India	ISE	[115]
~3	pour-over	5	1.5	150	nd	nd	nd	100	Arabica	roasted	fresh ground	Guatemala (Antigua region)	ISE	[115]
~2.2	Turkish coffee	5	1.5	150	nd	nd	nd	100	Arabica	roasted	fresh ground	Guatemala (Antigua region)	ISE	[115]
~2	French press	5	1.5	150	nd	nd	nd	100	Arabica	roasted	fresh ground	Guatemala (Antigua region)	ISE	[115]
~2	coffee percolator	5	1.5	150	nd	nd	nd	100	Arabica	roasted	fresh ground	Guatemala (Antigua region)	ISE	[115]
~2	filter coffee machine	5	1.5	150	nd	nd	nd	100	Robusta	roasted	fresh ground	India	ISE	[115]
~1.8	French press	5	1.5	150	nd	nd	nd	100	Robusta	roasted	fresh ground	India	ISE	[115]
~1	Turkish coffee	5	1.5	150	nd	nd	nd	100	Robusta	roasted	fresh ground	India	ISE	[115]

ISE—Fluoride ion-selective electrode.

## Data Availability

Not applicable.
